# NefrUS: ultrasonography in nephrology — an initiative of the Brazilian Society of Nephrology for ultrasound training in nephrology practice

**DOI:** 10.1590/2175-8239-JBN-2025-0331en

**Published:** 2026-03-06

**Authors:** Marcus Gomes Bastos, José A. Moura-Neto, Pedro Túlio Monteiro de Castro de Abreu Rocha

**Affiliations:** 1 Suprema – Faculdade de Ciências Médicas e da Saúde, Juiz de Fora, MG, Brazil.; 2 Centro Universitário Governador Ozanam Coelho, Ubá, MG, Brazil.; 3 Escola Bahiana de Medicina e Saúde Pública, Salvador, BA, Brazil.; 4 Redes das Américas, Hospital São Lucas, Rio de Janeiro, RJ, Brazil.; 5 Universidade Federal do Rio de Janeiro, Rio de Janeiro, RJ, Brazil.

Dear Editor,

Point-of-care ultrasonography (POCUS) has become an essential diagnostic tool across multiple medical specialties, allowing clinicians to integrate anatomical and functional findings at the bedside in real time^1^. In nephrology, POCUS is increasingly used for hemodynamic assessment, the management of acute and chronic kidney disease, renal transplantation, dialysis optimization, and the safe performance of invasive procedures^2^.

Despite its recognized clinical value, opportunities for POCUS training remain limited in nephrology education in Brazil. A national survey of members of the Brazilian Society of Nephrology (BSN) indicated that most nephrologists lacked formal ultrasound training, although nearly all expressed interest in acquiring POCUS skills for nephrology practice^3^. This gap highlights the urgent need for structured educational initiatives.

Aligned with recent recommendations from the International Alliance for POCUS in Nephrology^4^, the BSN established NefrUS – Ultrasonography in Nephrology, an educational program developed for residents, preceptors, and practicing nephrologists. The curriculum covers fundamental principles and clinical applications of POCUS in nephrology, preparing physicians to perform rapid, accurate assessments across diverse clinical environments, including outpatient clinics, hospital wards, dialysis units, and intensive care settings.

NefrUS uses a flipped classroom model to reduce cognitive load and enhance learning. The program combines pre-recorded theoretical lectures with supervised hands-on sessions, totaling eight hours of practical training. Each training station is staffed by one instructor, with three to four participants sharing a dedicated ultrasound machine. This setup facilitates individualized feedback and immediate supervised learning. The curriculum (Figure 1) covers image optimization; focused cardiac and pulmonary ultrasound; urinary tract imaging; ultrasound-guided invasive pro­cedures, such as central venous access and renal biopsy; and ultrasound applications for evaluating volume tolerance and fluid therapy response, especially in patients with acute kidney injury.

**Figure 1:**
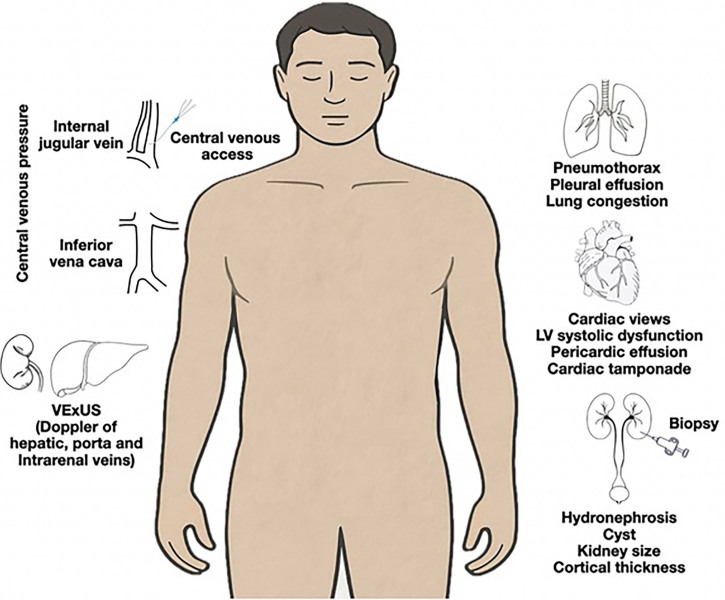
Topics addressed in the NefrUS hands-on sessions: image optimization; focused cardiac and pulmonary ultrasound; urinary tract imaging; ultrasound-guided invasive procedures, such as central venous access and renal biopsy; and ultrasound applications for evaluating volume tolerance and fluid therapy response, especially in patients with acute kidney injury.

Beyond technical proficiency, NefrUS reflects the BSN’s commitment to patient safety, image-based clinical reasoning, and continuing medical education. The standardization of POCUS instruction in nephrology represents a significant step toward integrated, contemporary, and evidence-based practice. Additionally, the program fosters professional networking to support the development of institutional protocols and collaborative training in nephrology ultrasonography.

As O’Neill and Ross^5^ emphasized, “We should not continue to practice nephrology and train future nephrologists the same way we did 25 years ago. Point-of-care ultrasound is rapidly becoming a standard component of patient care, and failure to embrace it will leave us further behind, akin to physicians who never adopted the stethoscope.” This vision summarizes the essence of NefrUS, which aims to establish POCUS as a natural extension of the nephrologist’s physical examination, fostering safer, more precise, and patient-centered care.

## References

[1] Díaz-Gómez JL, Mayo PH, Koenig SJ (2021). Point-of-care ultrasonography. N Engl J Med.

[2] Reisinger NC, Koratala A (2022). Incorporating training in POCUS in nephrology fellowship curriculum. Clin J Am Soc Nephrol.

[3] Bastos MG, Vieira AL, Nascimento MMD, Barros E, Pazeli JM, Kirsztajn GM (2021). Point-of-care ultrasonography in nephrology: a cross-sectional national survey among Brazilian nephrologists. J Bras Nefrol.

[4] Koratala A, Argaiz ER, Romero-González G, Reisinger N, Anwar S, Beaubien-Souligny W (2024). Point-of-care ultra­sound training in nephrology: a position statement by the International Alliance for POCUS in Nephrology. Clin Kidney J.

[5] O’Neill WC, Ross DW (2019). Retooling nephrology with ultrasound. Clin J Am Soc Nephrol.

