# Supplementary material for: NefrUS: ultrasonography in nephrology — an initiative of the Brazilian Society of Nephrology for ultrasound training in nephrology practice
Source: J Bras Nefrol. 2026 Mar 6;48(1):e20250331. doi: 10.1590/2175-8239-JBN-2025-0331en (PMC12965625; doi:10.1590/2175-8239-JBN-2025-0331en)
Supplement: Translation

# Translation: NefrUS: ultrassonografia em nefrologia — a iniciativa da Sociedade Brasileira de Nefrologia para formação em ultrassonografia *point-of-care* na prática nefrológica

**DOI:** 10.1590/2175-8239-JBN-2025-0331pt

**Authors:** Marcus Gomes Bastos, José A. Moura-Neto, Pedro Túlio Monteiro de Castro de Abreu Rocha

**Affiliations:** 1 Suprema – Faculdade de Ciências Médicas e da Saúde, Juiz de Fora, MG, Brasil.; 2 Centro Universitário Governador Ozanam Coelho, Ubá, MG, Brasil.; 3 Escola Bahiana de Medicina e Saúde Pública, Salvador, BA, Brasil.; 4 Redes das Américas, Hospital São Lucas, Rio de Janeiro, RJ, Brasil.; 5 Universidade Federal do Rio de Janeiro, Rio de Janeiro, RJ, Brasil.

Prezado Editor,

A ultrassonografia *point of care* (POCUS) consolidou-se, nas últimas décadas, como ferramenta diagnóstica essencial em diversas especialidades médicas, permitindo ao clínico integrar achados anatômicos e funcionais à beira do leito, em tempo real^1^. Na nefrologia, a POCUS tem papel crescente na avaliação hemodinâmica, no manejo da injúria renal aguda e crônica, no transplante renal, na otimização da diálise e na realização de procedimentos invasivos com maior segurança^2^.

Entretanto, a incorporação dessa competência à formação do nefrologista ainda é incipiente no Brasil. Em levantamento nacional realizado entre os sócios da Sociedade Brasileira de Nefrologia (SBN), verificou-se que a maioria dos nefrologistas entrevistados não foi treinada em ultrassonografia; contudo, a quase totalidade dos participantes da pesquisa manifestou interesse em aprender a utilizar o POCUS na prática nefrológica^3^. Esses dados revelam uma lacuna importante na formação e ressaltam a necessidade de iniciativas estruturadas de capacitação.

Em consonância com as recomendações recentes da *International Alliance for POCUS in Nephrology* ^4^, a SBN lançou o curso NefrUS — Ultrassonografia em Nefrologia, uma iniciativa pioneira voltada à capacitação de residentes, preceptores e nefrologistas em atividade clínica. O NefrUS tem como proposta introduzir os princípios e as aplicações fundamentais da POCUS no contexto nefrológico, habilitando o médico a realizar avaliações rápidas e acuradas em diferentes cenários assistenciais — que vão do consultório, da enfermaria e da unidade de diálise à unidade de terapia intensiva.

Com o objetivo de reduzir a sobrecarga cognitiva no processo de ensino-aprendizagem, o NefrUS foi estruturado segundo o modelo de *flipped classroom*, combinando videoaulas teóricas e atividades práticas supervisionadas, totalizando oito horas de treinamento presencial. Cada estação mantém a proporção de um instrutor, um equipamento e três a quatro participantes, assegurando feedback individualizado e aprendizado supervisionado imediato. A ementa (Figura 1) contempla o ajuste do ultrassom para a obtenção de imagens de qualidade; a ultrassonografia focada do coração; o POCUS pulmonar; a insonação do trato urinário; procedimentos invasivos guiados por ultrassonografia (acesso venoso central e biópsia renal); e a POCUS na avaliação da tolerância e da resposta à fluidoterapia, bem como no paciente com injúria renal aguda.

**Figura 1:** Temas abordados no NefrUS: ajuste do ultrassom para a obtenção de imagens de qualidade; ultrassonografia focada do coração; POCUS pulmonar; insonação do trato urinário; procedimentos invasivos guiados por ultrassonografia (acesso venoso central e biópsia renal); e POCUS na avaliação da tolerância e da resposta à fluidoterapia, bem como no paciente com injúria renal aguda.

Além de oferecer formação técnica, o NefrUS reforça o compromisso da SBN com a segurança assistencial, o raciocínio clínico estendido com imagem e a educação médica continuada. A padronização do ensino da POCUS em nefrologia representa um passo decisivo para uma prática mais integrada, moderna e alinhada às demandas da medicina baseada em evidências. O curso também constitui uma oportunidade de congregar profissionais interessados em desenvolver protocolos institucionais e redes colaborativas em ultrassonografia aplicada à nefrologia.

Como destacaram os doutores O’Neill e Ross^5^, “a nefrologia não deve ser praticada nem ensinada como há 25 anos. O POCUS tornou-se parte essencial do cuidado clínico, e ignorá-lo seria como ter recusado o estetoscópio. Incorporar essa abordagem à formação em nefrologia é fundamental para aprimorar o cuidado aos pacientes e atrair novos profissionais para a especialidade.” Essa visão sintetiza a essência do NefrUS, que busca consolidar a POCUS como extensão natural do exame físico do nefrologista, promovendo um cuidado mais preciso, seguro e centrado no paciente.
